# Long-Term Quercetin Dietary Enrichment Partially Protects Dystrophic Skeletal Muscle

**DOI:** 10.1371/journal.pone.0168293

**Published:** 2016-12-15

**Authors:** Hannah R. Spaulding, Christopher G. Ballmann, John C. Quindry, Joshua T. Selsby

**Affiliations:** 1 Department of Animal Science, Iowa State University, Ames, IA, United States of America; 2 School of Kinesiology, Auburn University, Auburn, AL, United States of America; University of Louisville School of Medicine, UNITED STATES

## Abstract

Duchenne muscular dystrophy (DMD) results from a genetic lesion in the dystrophin gene and leads to progressive muscle damage. PGC-1α pathway activation improves muscle function and decreases histopathological injury. We hypothesized that mild disease found in the limb muscles of mdx mice may be responsive to quercetin-mediated protection of dystrophic muscle via PGC-1α pathway activation. To test this hypothesis muscle function was measured in the soleus and EDL from 14 month old C57, mdx, and mdx mice treated with quercetin (mdxQ; 0.2% dietary enrichment) for 12 months. Quercetin reversed 50% of disease-related losses in specific tension and partially preserved fatigue resistance in the soleus. Specific tension and resistance to contraction-induced injury in the EDL were not protected by quercetin. Given some functional gain in the soleus it was probed with histological and biochemical approaches, however, in dystrophic muscle histopathological outcomes were not improved by quercetin and suppressed PGC-1α pathway activation was not increased. Similar to results in the diaphragm from these mice, these data suggest that the benefits conferred to dystrophic muscle following 12 months of quercetin enrichment were underwhelming. Spontaneous activity at the end of the treatment period was greater in mdxQ compared to mdx indicating that quercetin fed mice were more active in addition to engaging in more vigorous activity. Hence, modest preservation of muscle function (specific tension) and elevated spontaneous physical activity largely in the absence of tissue damage in mdxQ suggests dietary quercetin may mediate protection.

## Introduction

Duchenne muscular dystrophy (DMD) is caused by the absence of the dystrophin protein, which acts to transmit force between cytoskeleton and extracellular matrix via the dystrophin glycoprotein complex (DGC) [1, 2]. The absence of dystrophin results in cellular dysfunction including decreased calcium homeostasis, increased necrosis, and disruption of DGC along with other secondary effects producing whole muscle dysfunction. Utrophin, a dystrophin-like protein, participates in DGC formation, stability, and function in the absence of dystrophin [3, 4], hence utrophin upregulation remains an area of intense research interest [5–8]. Utrophin transcription can be driven by the exercise-sensitive PGC-1α pathway [9], however, attempts to use various exercise modalities as interventions for DMD have been met with mixed results [10–13]. Direct activation of the PGC-1α pathway using transgenic and gene transfer approaches yields consistently positive results using both prevention and rescue paradigms [14–18]. Under these conditions PGC-1α pathway activation led to increased muscle function, decreased muscle damage, increased utrophin abundance and a physiologic and metabolic type I shift in PGC-1α over-expressing dystrophic muscle compared to control muscle [14–18].

Given the emerging success of PGC-1α pathway activation for treating dystrophic pathology we next searched for PGC-1α activators that already had FDA approval or were freely available to minimize time needed to impact patients. Quercetin, a flavonoid with antioxidant and anti-inflammatory properties [19], drives the PGC-1α pathway through SIRT1 deacetylase [20, 21] or AMPK activity [22]. We found previously that six months of dietary quercetin enrichment decreased histopathology in diaphragms [23] and hearts [24] from dystrophic mice. In a follow up experiment we found that 12 months of quercetin dietary enrichment transiently protected dystrophic diaphragms and respiratory function though a developed quercetin insensitivity ultimately minimized therapeutic benefits [25]. Given that limb muscles from mdx mice suffer a more mild disease than diaphragms we reasoned that quercetin may continue to protect limb muscle from progressive disease and, therefore, that quercetin would have a role as a therapeutic intervention early in the disease process and would be most efficacious in the youngest DMD patients. We hypothesized that muscle function would be improved and histological injury would be decreased in dystrophic soleus and extensor digitorum longus (EDL) following 12 months of dietary quercetin enrichment compared to muscles taken from mice maintained on a control diet.

## Methods

### Ethical Approval and Animal Treatments

The Institutional Animal Care and Use Committee at Auburn University reviewed and approved all procedures utilized in this work. Previous work, including a detailed study design, has been previously published [25]. Briefly, eight male C57 mice and 16 male mdx mice (Jackson Laboratories) were acclimated for one week prior to the beginning of the experiments. At 2 months of age a standard AIN93 diet (Bioserv, Flemington, NJ) was provided for C57 mice (n = 8) and control mdx mice (n = 8), while treated mdx mice received an AIN93 diet supplemented with 0.2% quercetin (n = 8) for 12 months. Both water and food were available *ad libitum*. Throughout the study period animal food, water, bedding, general health, and environmental conditions were checked twice daily by a combination of research and vivarium staff. Over the course of this longitudinal investigation one mouse from each group was identified as severely ill (monitored criteria included: physical appearance, weight loss, and behavior). To minimize suffering animals were euthanized via CO2 inhalation followed by exsanguination consistent with our IACUC protocol. Changes in body weight throughout the study period have been previously reported as have average daily food consumption (3.9 g/day) and resultant daily quercetin exposure (204 mg/kg/day) [25]. At 14 months of age, activity was measured using an ethiological approach. Soleus and EDL *in vitro* muscle function were assessed at the Physiological Assessment Core of the Wellstone Muscular Dystrophy Cooperative Center at the University of Pennsylvania. Prior to *in vitro* function all animals were assigned new numbers to establish blinded data collection and further preserve blinded conditions upon distribution of tissues for subsequent analyses. Sample numbers and animal groups were revealed prior to biochemical analysis for properly controlled experiments. Due to the length of this study several animals did not reach 14 months of age or tissues were unusable for some measures, thus number/group is identified for all measurements in the figure legends.

### Animal Activity

At 14 months of age, activity was recorded for 10 consecutive minutes in conscious mice and were averaged over two observation periods for occurrences of sitting, grooming, eating/drinking, socializing, standing, walking, wall pacing, running, and jumping. This technique represents a species-specific ethogram (repertoire of discrete animal activities) using the “0–1 recording” method, which has been applied across many species. When an observed mouse performed a particular activity (sitting, walking, etc.) it was recorded as 1. Activity counts were performed on two occasions by two investigators and activity recordings were performed every 15 seconds for 10 minutes and collectively equaled 40 activity time periods. Twenty total minutes of activity were recorded for the two sessions and final counts were averaged from scores generated by the two blinded observers. Activity counts were performed at a common time at the end of the photo light and photo dark cycles [26].

### Tissue Collection and Muscle Function

After 12 months of treatment, mice were sedated to a surgical level of anesthesia using a ketamine/xylazine cocktail at the Physiological Assessment Core of the Wellstone Muscular Dystrophy Cooperative Center at the University of Pennsylvania (now housed at the University of Florida). Upon sedation, soleus and EDL muscles were removed from each animal and used for measures of muscle function. *In vitro* muscle function was measured using standard techniques as has been done previously [27–29]. Briefly, the tetanic force was determined in the EDL and soleus using stimulation of 120 Hz and 100 Hz, respectively. To determine fatigue resistance the soleus was stimulated for 10 minutes with one contraction/sec (100 Hz for 330 msec with 200 μsec pulse). To determine resistance to contraction-induced injury the EDL was given a series of five lengthening contractions (500 msec at 80 hz followed by 10% beyond Lo for 20 msec). To determine fatigue resistance and resistance to contraction induced injury data are expressed relative to peak force produced during the first contraction. Specific tension and cross sectional area were calculated using standard equations [30]. As there were changes in soleus muscle function suggestive of a therapeutic effect tissues were examined with histological and biochemical approaches. Following the fatigue test the soleus was frozen in melting isopentane and used for histological measures while the contralateral soleus was snap frozen upon removal and used for biochemical measures.

### Histological Analyses

Muscle injury and fibrosis were measured as recently described [25]. Briefly, 10 μm sections were cut and stained with either hematoxylin and eosin (H&E) or Masson’s Trichrome (KTMTR, American MasterTech, Lodi, CA). H&E and trichrome stained slides were imaged with an inverted DMI3000 B microscope and QICAM MicroPublisher 5.0 (MP5.0-RTV-CLR-10, QIMAGING) camera using QCapture software. Trained, blinded technicians took 3–5 images at 10x magnification for each soleus section. Overlapping images allowed for reconstruction of the entire muscle cross section using the Photoshop merge option (Adobe). H&E sections were then analyzed by these technicians using Open Lab (Improvision) to quantify 1) total number of muscle cells, 2) central nucleation, 3) extracellular nuclei, 4) necrotic area, and 5) total contractile area (area of the section comprised of muscle fibers). Trichrome staining was used to quantify areas of fibrosis.

Immunohistochemistry was utilized to confirm fibrosis findings and measure fiber area distribution. To measure fibrosis and fiber area distribution anti-fibronectin (Sigma, St. Louis, MO) and anti-laminin (1:100, Thermo Scientific, Waltham, MA) primary antibodies were applied to the samples at 4°C overnight, respectively. At room temperature, sections were exposed to donkey anti-rabbit rhodamine secondary antibody (1:200, Millipore, Billerica, MA) for 1 hour. 3–5 non-overlapping images were taken with a QICAM 12-bit Mono Fast 1394 Cooled (QIC-F-M-12-C, QIMAGING) camera at 10x magnification attached to the Leica microscope under blinded conditions.

### qPCR

Measurement of transcript abundance was performed in the soleus employing Fluidigm technology as we have done previously [25]. Our complete list of primer pairs has also been previously published. Briefly, mRNA was isolated using TriZol (ThermoScientific) and reverse transcribed to cDNA using QuantiTect Reverse Transcriptase Kit (Qiagen) as described by the manufacture, but random hexamers (IDT PreMade Primers) were substituted for the RT Primer Mix (Qiagen). cDNA was further prepared as suggested by Fluidigm then loaded onto a 96x96 Fluidigm chip.

### Western Blot

Measurement of relative protein abundance was performed as previously described. Briefly, 200 μl of whole muscle buffer (10mM Sodium Phosphate buffer, pH 7.0, 2% SDS) was added to powdered soleus tissue and homogenized. Once homogenized, samples were spun at 20,000 RCF for 15 minutes and supernatant containing the protein was collected for western blotting. Protein concentration was measured using Pierce BCA Protein Assay Kit (ThermoScientific, 23225) and diluted with 2x Laemmli buffer. Once diluted, samples were heated for 5 minutes at 95°C. Thirty micrograms of protein were separate by mass using 4–20% gradient gels (Lonza). Separation was run at 60 volts for 20 minutes followed by 120 volts for 60 minutes, then transferred for 60 minutes at 100 volts onto a nitrocellulose membrane. Membranes were probed for the following antibodies overnight at 4°C then incubated for 1 hour with anti-rabbit secondary: phosphorylated (p-) AMPKα (thr172) (P 1:500, S 1:2000, Cell Signaling), SIRT1 (P 1:500, S 1:2000, Millipore), Histone 3 Lysine 9 Acetylation (H3K9ac) (P 1:1000, S 1:5000 in 0% milk, Cell Signaling), ERRα (P 1:1000, S 1:2000), TFAM (P 1:375 in 1% milk, S 1:1000 in 0% milk), Cytochrome C (P 1:1000, S 1:2000), SDHA (P 1:500, S 1:2000), VDAC (P 1:300, S 1:2000).

### Statistics

All data were assessed using one-way ANOVA with a Newman-Keuls post-hoc test to assess significance of p<0.05. Unless otherwise noted, data are shown as mean ± SEM.

## Results

To determine the extent to which quercetin decreased dystrophic injury mdx mice were treated with a 0.2% quercetin enriched diet or maintained on a control diet from 2–14 months of age. We have previously reported detailed food consumption and growth data [25]. In brief, animal growth was largely similar between groups though the mdxQ group was statistically smaller than the mdx group at the conclusion of the investigation (C57–44.07 ± 2.13 g, mdx– 56.57 ± 2.30 g, mdxQ– 38.07 ± 2.35 g). In the soleus, dystrophin-deficiency caused a 40% increase in absolute muscle mass regardless of treatment (Table 1). Relative muscle mass was nearly doubled in mdx compared to C57 and mdxQ was 11% greater (p<0.05) than mdx (Table 1). In the EDL, absolute mass was 30 and 13% greater in mdx compared to C57 and mdxQ, respectively, and mdxQ was 20% greater than C57 (Table 1). Relative EDL mass in dystrophic mice was increased by 60% compared to C57 (Table 1). Data for all measures is included in S1 Table.

**Table 1:** Soleus and EDL muscle weights and function. * indicates significantly different from C57; # indicates significantly different from mdx. C57 (n = 6–7) mdx (n = 6) mdxQ (n = 5–6).

	Soleus			EDL		
	C57	mdx	mdxQ	C57	Mdx	mdxQ
**Mass (mg)**	11.39 ± 0.17	17.61 ± 0.54 *	15.96 ± 1.08 *	12.73 ± 0.31	18.65 ± 0.67 *	16.30 ± 0.76 *^#^
**Relative Mass (mg/g)**	0.25 ± 0.01	0.45 ± 0.01 *	0.49 ± 0.02 *^#^	0.28 ± 0.01	0.47 ± 0.01 *	0.47 ± 0.03 *
**Tetanic Force (mN)**	224.7 ± 8.1	209.6 ± 21.5	252.9 ± 8.8	506.8 ± 13.7	461.1 ± 33.4	451.2 ± 13.0

### *In vitro* function

**Fig 1:**
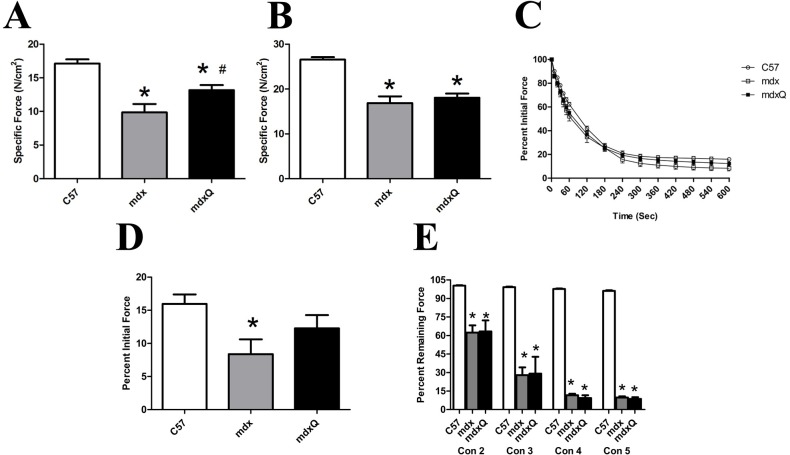
Soleus and EDL muscle function at 14 months of age. A) Specific tension measured in the soleus and B) in the EDL. C) The soleus was then subjected to a fatigue test where it was stimulated once per second for 10 minutes. Force was normalized to force produced in the first contraction. D) The relative force produced in the final contraction is shown. Relative force produced in the final contraction was significantly less in mdx than C57. E) The EDL was subjected to a series of five eccentric contractions and peak force normalized to peak force generated in the first contraction. * indicates significantly different from C57; # indicates significantly different from mdx. C57 (n = 7) mdx (n = 6) mdxQ (n = 6).

Tetanic force was similar between groups in the soleus. Specific tension, however, was decreased by 40% in mdx compared to C57. Importantly, dietary quercetin enrichment attenuated approximately 50% of this loss (Fig 1). Following a fatigue protocol, the percent of initial force produced by the mdx soleus was decreased by 50% compared to C57. The addition of quercetin appeared to attenuate this loss but this numerical difference was not statistically significant from the other treatments (Fig 1C).

Consistent with the soleus muscle, tetanic tension in the EDL was similar between groups (Table 1). Specific tension was decreased by 36% in mdx and mdxQ groups compared to C57 (Fig 1B). In addition, dietary quercetin failed to provide protection from eccentric injury in the EDL as the percent decline was similar in mdx and mdxQ and considerably less than C57 for each contraction (Fig 1E).

**Fig 2:**
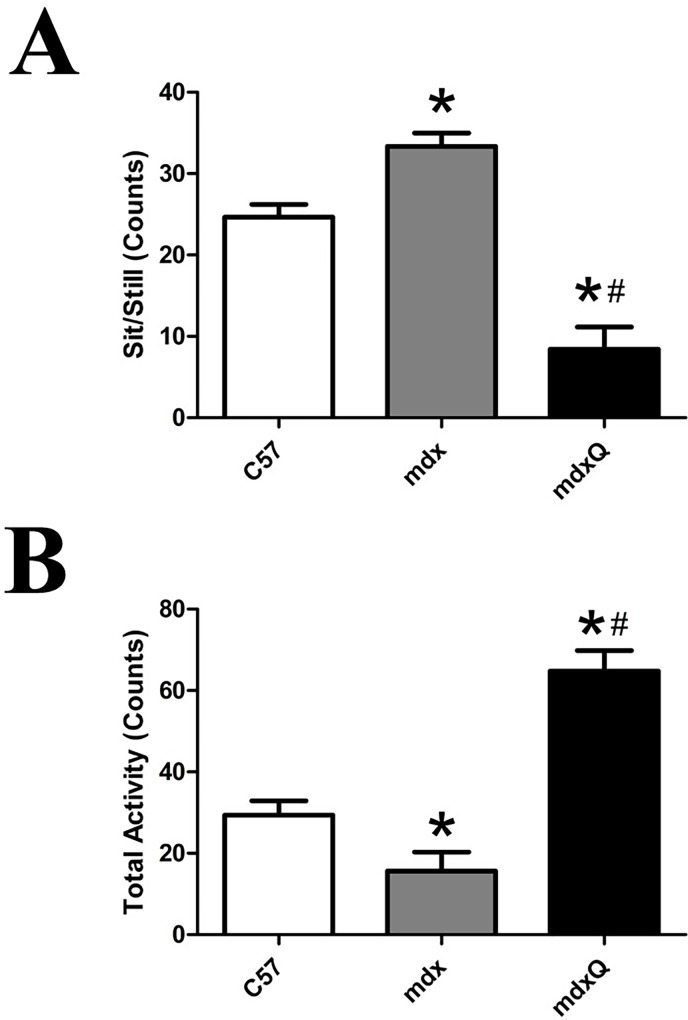
Animal activity was increased by dietary quercetin enrichment. At the conclusion of the investigation animal behavior was quantified using an ethological approach where a 10 minute observation period was divided into 15 second blocks. A) The number of time blocks spent sitting or exhibiting sedentary behavior was quantified. B) We also quantified the number of active behaviors. * indicates significantly different from C57; # indicates significantly different from mdx. C57 (n = 7) mdx (n = 7) mdxQ (n = 6).

Animal activity was assessed by counts of sedentary and active behaviors during 15 second sampling windows averaged over two 10 minute observation periods. For the sitting/standing metrics, mdx mice were 35% more stationary than C57 and mdxQ was less stationary than both C57 and mdx mice by 65% and 74%, respectively (Fig 2A). Total activity followed a similar pattern such that mdx were 47% less active than C57 whereas mdxQ were 2-fold more active than C57 (Fig 2B).

### Histopathology

**Fig 3:**
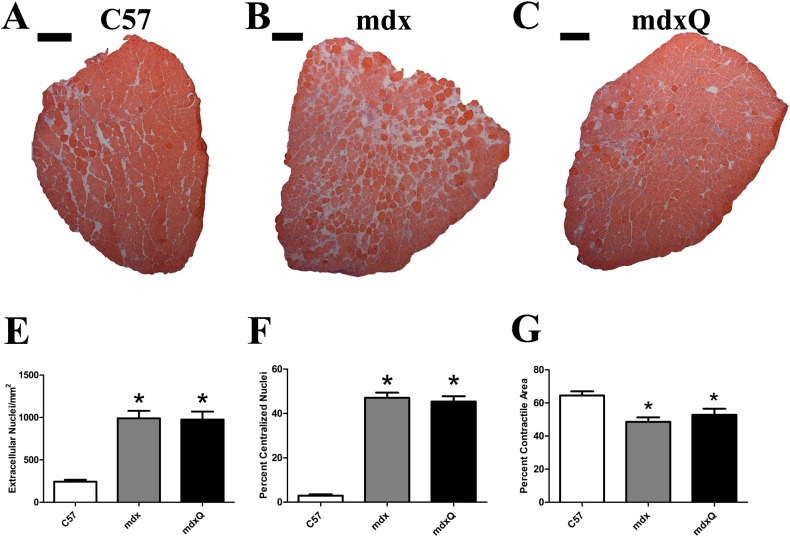
Dystrophin deficiency causes histological injury that is not corrected by quercetin. A-C) Representative images from H&E-stained, reconstructed soleus muscle cross sections. E) Extra cellular nuclei, F) centralized nuclei, and G) total contractile area were calculated. * indicates significantly different from C57. Width of black bar represents 250 microns. C57 (n = 7) mdx (n = 6) mdxQ (n = 6).

**Fig 4:**
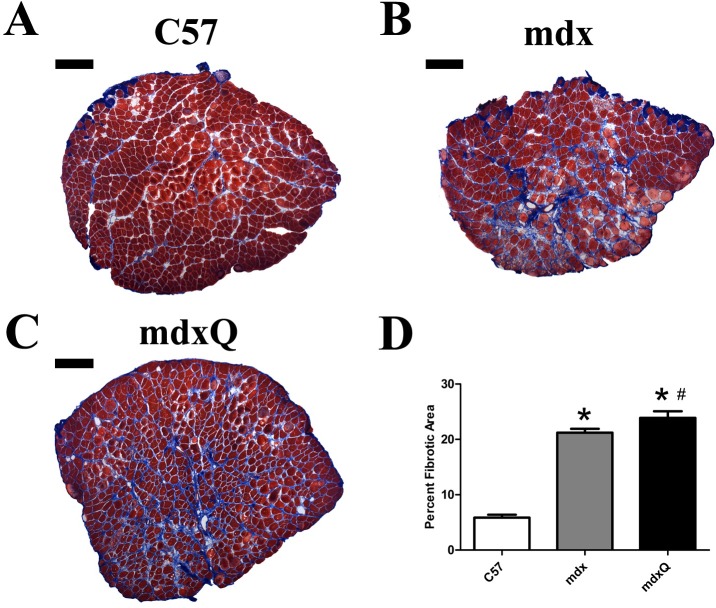
Dystrophin deficiency increased muscle fibrosis. A-C) Representative images from trichrome-stained, reconstructed soleus cross sections. D) Total fibrotic area was calculated by quantifying the blue staining material in the entire muscle cross section. * indicates significantly different from C57. Width of black bar represents 250 microns. C57 (n = 7) mdx (n = 6) mdxQ (n = 6).

**Fig 5:**
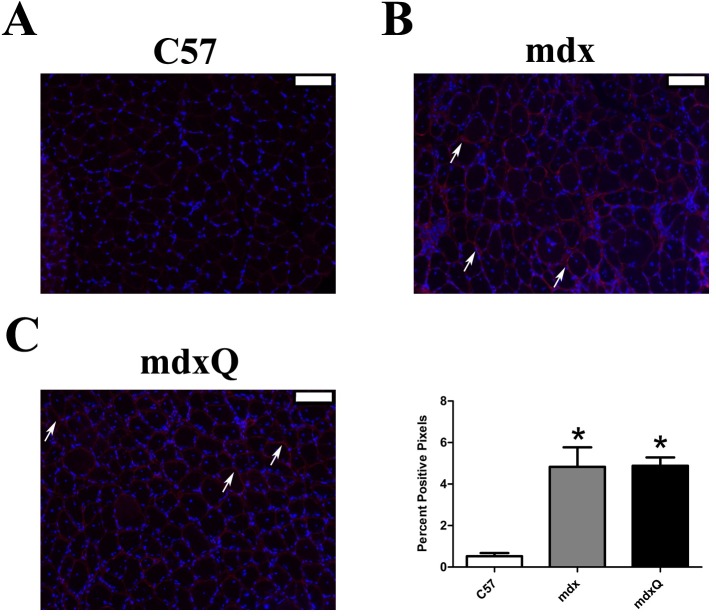
Dystrophin deficiency increased fibronectin in soleus muscles compared to healthy muscles. A-C) Representative 10x immunohistochemical images for fibronectin (red) and DAPI (blue). All images have been uniformly brightened to make the fibronectin signal easier to see. D) The percent positive pixels were quantified. * indicates significantly different from C57. Width of white bar represents 100 microns. C57 (n = 7) mdx (n = 6) mdxQ (n = 6).

Given that improved specific tension and fatigue resistance in the soleus provided some limited optimism for a successful intervention histological and biochemical studies were pursued. The quantity of extracellular nuclei was increased by 4-fold and the percent of cells with a centralized nucleus was increased by 15-fold in dystrophic muscle compared to healthy muscle and both measures were resistant to quercetin (Fig 3A–3F). Consistent with disease-related injury the total contractile area was decreased 20% in mdx and mdxQ mice compared to C57 due to an accumulation of non-contractile material in the whole muscle cross-section (Fig 3G). Fibrotic tissue in the soleus of mdx mice measured via trichrome staining was 3.6-fold higher while mdxQ mice were 4-fold higher than C57. In addition, trichrome staining revealed that fibrosis in mdxQ increased by 12.5% compared to mdx (Fig 4A–4D). To verify these changes we also assessed relative fibronectin abundance using an immunohistochemical approach. We found that fibronectin was 9.0-fold higher in both mdx and mdxQ compared to C57 and importantly, that mdx and mdxQ were similar between groups (Fig 5A–5D).

**Fig 6:**
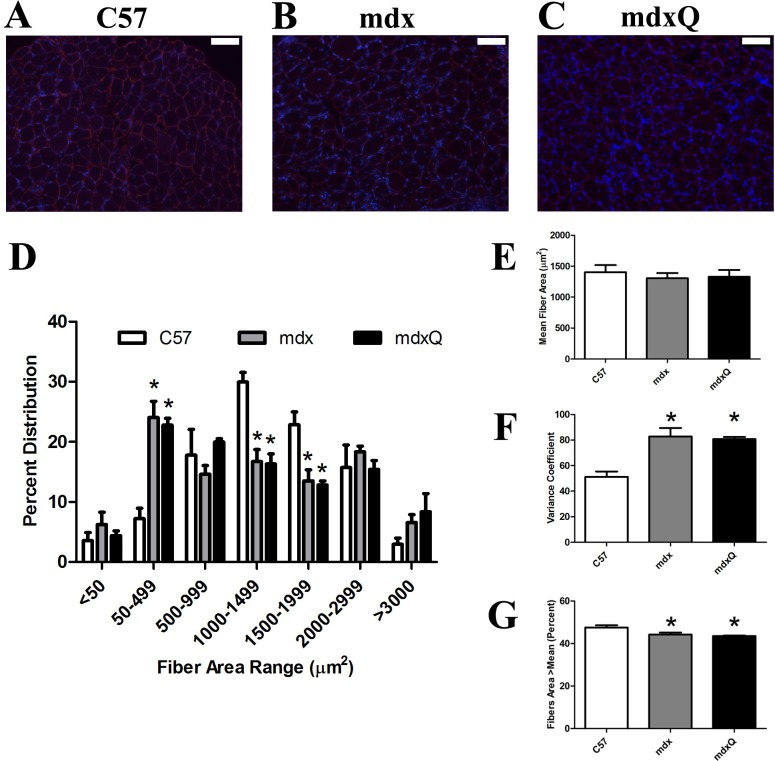
Fiber area distribution is altered by dystrophin deficiency in soleus muscle. A-C) Representative 10x images from an immunohistological experiment where laminin (red) was detected. DAPI is shown in blue. D) Fiber area distribution was measured and quantified in bins. E) Mean fiber area and F) the variance coefficient were calculated. G) We also determined the percent of fibers greater than the mean cross sectional area as another indicator of fiber area variability. * indicates significantly different from C57. Width of white bar represents 100 microns. C57 (n = 7) mdx (n = 6) mdxQ (n = 6).

Lastly, we also measured fiber area distribution (Fig 6A–6D). We found that at 14 months of age mean fiber area was similar between groups, however, the variance coefficient was 40% higher in dystrophic muscle compared to healthy muscle (Fig 6E and 6F). Further, we found that dystrophic muscle had a greater frequency of smaller diameter fibers and a smaller frequency of larger diameter fibers compared to healthy muscle (Fig 6G). While dystrophic muscle tended to have a greater frequency of very large fibers (>3000 μm) this observation was not statistically significant from C57 (Fig 6D).

### Biochemistry

We also assessed the impact of dystrophin deficiency on alterations in transcript expression and the degree to which these were corrected by dietary quercetin enrichment (Table 2). Our findings largely suggest that following 12 months of quercetin treatment transcript expression was similar between mdx and mdxQ, however, numerous differences between dystrophic and healthy muscle were noted. Specifically, metabolic transcripts were significantly decreased in mdx mice compared to C57 as were transcripts related to mitochondrial biogenesis, redox balance, and cellular stability.

**Table 2:** Transcript expression in the soleus. Data are shown as fold change relative to C57. * indicates significantly different from C57; # indicates significantly different from mdx. C57 (n = 7) mdx (n = 6) mdxQ (n = 6).

	C57	Mdx	mdxQ
	Average ± SEM	Average ± SEM	Average ± SEM
**Mitochondrial Biogenesis (PGC-1α Pathway Genes)**			
Sirt1	1.00 ± 0.25	2.02 ± 0.45	1.94 ± 0.54
Ppargc1a (Pgc-1α)	1.00 ± 0.11	-1.67 ± 0.09*	-1.72 ± 0.09*
Esrra (Errα)	1.00 ± 0.18	-3.47 ± 0.11*	-2.65 ± 0.06*
Nrf1	1.00 ± 0.23	-1.93. ± 0.11	-1.24 ± 0.28
Nrip1	1.00 ± 0.18	-1.45 ± 0.20	-1.46 ± 0.21
Tfam	1.00 ± 0.48	-1.34 ± 0.16	-2.07 ± 0.11
**Metabolic Genes**			
Fnip1	1.00 ± 0.25	-1.84 ± 0.06	-2.08 ± 0.04
Mtor	1.00 ± 0.27	-2.48 ± 0.12*	-2.57 ± 0.10*
Prkaa1	1.00 ± 0.16	-1.05 ± 0.14	-1.72 ± 0.12
Gapdh	1.00 ± 0.20	-3.48 ± 0.04*	-2.97 ± 0.04*
Pfkm	1.00 ± 0.16	-2.38 ± 0.04*	-2.38 ± 0.06*
Pparg	1.00 ± 0.19	-1.32 ± 0.27	-1.18 ± 0.40
Cs	1.00 ± 0.16	-3.64 ± 0.05*	-3.60 ± 0.04*
Mdh1	1.00 ± 0.15	-1.94 ± 0.07*	-2.27 ± 0.08*
Atp1a2	1.00 ± 0.07	-1.76 ± 0.10*	-2.09 ± 0.06*
Cybb	1.00 ± 0.30	-1.35 ± 0.03	2.77 ± 1.35
Cycs	1.00 ± 0.27	1.11 ± 0.29	-2.45 ± 0.11
Mb	1.00 ± 0.17	-4.35 ± 0.03*	-4.61 ± 0.04*
Mt-atp6	1.00 ± 0.07	-2.14 ± 0.05*	-2.38 ± 0.06*
Mt-co1	1.00 ± 0.17	-2.78 ± 0.04*	-3.18 ± 0.05*
Mt-co2	1.00 ± 0.14	-2.15 ± 0.07*	-2.50 ± 0.06*
Mt-cyb	1.00 ± 0.09	-2.35 ± 0.04*	-2.59 ± 0.06*
Mt-nd1	1.00 ± 0.06	-2.32 ± 0.04*	-2.66 ± 0.06*
Mt-nd4	1.00 ± 0.21	-2.32 ± 0.05*	-2.85 ± 0.06*
Uqcrc1	1.00 ± 0.17	-2.01 ± 0.09*	-2.59 ± 0.05*
Tfb1m	1.00 ± 0.66	-1.49 ± 0.26	-2.95 ± 0.06
Tfb2m	1.00 ± 0.13	-2.50 ± 0.09*	-1.70 ± 0.16
Ucp3	1.00 ± 0.21	-3.50 ± 0.11*	-4.50 ± 0.04*
Ak1	1.00 ± 0.35	-2.68 ± 0.12	-2.18 ± 0.27
Akt1	1.00 ± 0.56	-4.43 ± 0.07	-2.97 ± 0.11
Ckm	1.00 ± 0.15	-1.87 ± 0.08	-1.92 ± 0.10
**Inflammation/antioxidant Genes**			
Nfĸb1	1.00 ± 0.24	-1.35 ± 0.31	-1.10 ± 0.40
Il1b	1.00 ± 0.28	-1.23 ± 0.21	-2.55 ± 0.02
Tlr4	1.00 ± 0.61	-2.18 ± 0.19	-3.56 ± 0.06
Traf2	1.00 ± 0.16	-1.02 ± 0.40	1.23 ± 0.32
Cat	1.00 ± 0.51	-5.53 ± 0.03*	-4.96 ± 0.06*
Gpx1	1.00 ± 0.47	-2.27 ± 0.17	-3.64 ± 0.07
Gpx4	1.00 ± 0.31	-2.29 ± 0.09*	-2.69 ± 0.05*
Gsr	1.00 ± 0.29	2.02 ± 0.93	-1.25 ± 0.17
Nfe2l2	1.00 ± 0.15	-1.98 ± 0.13*	-2.11 ± 0.13*
Prdx2	1.00 ± 0.25	-2.96 ± 0.06*	-4.00 ± 0.04*
Sod1	1.00 ± 0.40	-2.36 ± 0.10	-3.37 ± 0.05*
Sod2	1.00 ± 0.25	-2.64 ± 0.05*	-2.88 ± 0.06*
**Apoptosis Genes**			
Apaf1	1.00 ± 0.27	-1.03 ± 0.07	1.68 ± 0.68
Bax	1.00 ± 0.37	1.12 ± 0.22	1.16 ± 0.28
Bcl2	1.00 ± 0.13	-1.59 ± 0.18	-1.63 ± 0.22
Bnip2	1.00 ± 0.17	-2.45 ± 0.08*	-1.29 ± 0.12*^#^
Casp3	1.00 ± 0.44	-1.29 ± 0.33	-2.10 ± 0.18
Trp53	1.00 ± 0.33	-2.97 ± 0.14	-1.19 ± 0.47
Xiap	1.00 ± 0.27	-2.05 ± 0.12	-1.92 ± 0.12
**Muscle Repair and Protein Turnover Genes**			
Fbl	1.00 ± 0.35	-1.48 ± 0.21	-1.15 ± 0.26
Gata2	1.00 ± 0.60	-2.16 ± 0.19	-3.78 ± 0.12
Hspa1a	1.00 ± 0.87	-1.88 ± 0.03	-2.47 ± 0.02
Hspa5	1.00 ± 0.36	-2.60 ± 0.07*	-1.97 ± 0.11
Hspb1	1.00 ± 0.21	-1.33 ± 0.18	-1.99 ± 0.11*
Mef2c	1.00 ± 0.17	-1.96 ± 0.09*	-2.68 ± 0.03*
Mstn	1.00 ± 0.26	-1.35 ± 0.19	-1.20 ± 0.21
Myf5	1.00 ± 0.38	-3.32 ± 0.08	-1.39 ± 0.38
Myocd	1.00 ± 0.26	-1.77 ± 0.33	-1.13 ± 0.71
Myod1	1.00 ± 0.67	-1.33 ± 0.53	-2.48 ± 0.15
Myof	1.00 ± 0.58	-2.06 ± 0.19	-2.39 ± 0.15
Myog	1.00 ± 0.59	-2.24 ± 0.17	-4.87 ± 0.07
Poldip2	1.00 ± 0.16	-1.24 ± 0.17	-2.11 ± 0.03*
Tgfb1	1.00 ± 0.10	1.45 ± 0.47	2.10 ± 1.00
**Structural and Sarcomeric Genes**			
Dag1	1.00 ± 0.10	-1.53 ± 0.13*	-2.27 ± 0.07*
Dtna	1.00 ± 0.16	-1.43 ± 0.08	-1.40 ± 0.12
Dysf	1.00 ± 0.16	-1.40 ± 0.15	-1.58 ± 0.10
Myh1	1.00 ± 0.39	-1.22 ± 0.15	-1.78 ± 0.05
Myh2	1.00 ± 0.17	-3.24 ± 0.04*	-3.29 ± 0.06*
Myh7	1.00 ± 0.17	-2.56 ± 0.05*	-2.92 ± 0.09*
Sgca	1.00 ± 0.17	-2.74 ± 0.05*	-3.49 ± 0.06*
Utrn 3’	1.00 ± 0.24	-1.15 ± 0.15	-1.32 ± 0.22
Utrn 5’	1.00 ± 0.15	-1.54 ± 0.14	-1.17 ± 0.32

**Fig 7:**
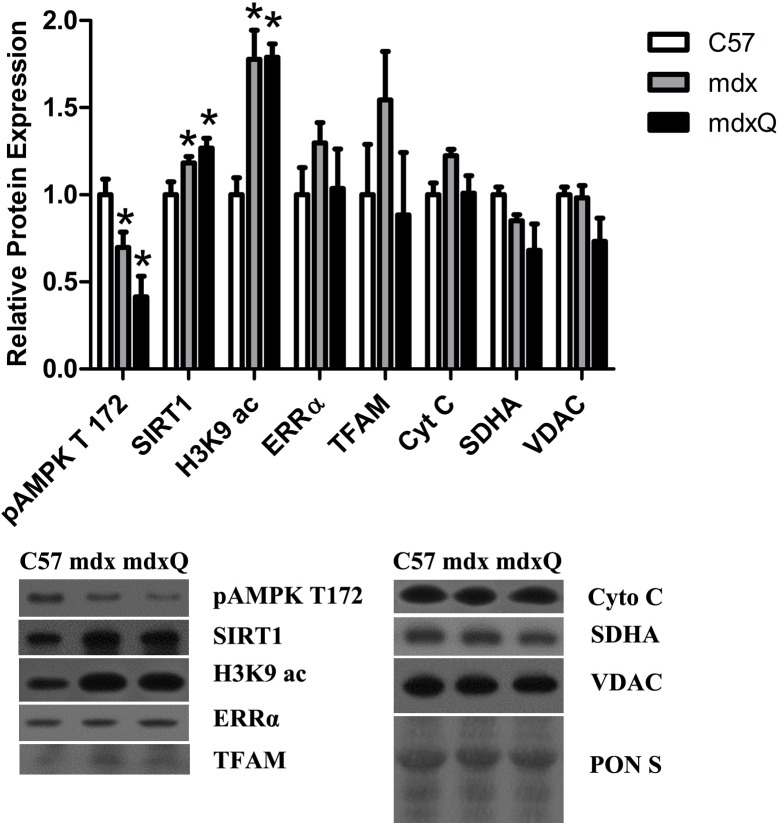
Relative protein abundance was altered by dystrophin deficiency. A) Protein abundance of PGC-1α pathway components was largely depressed by dystrophin deficiency independent of intervention compared to muscle from C57 mice. B) Representative blots are included and Ponceau S stain is included to demonstrate equal loading. * indicates significantly different from C57. C57 (n = 5) mdx (n = 6) mdxQ (n = 5).

Lastly, we evaluated the pathway driven by quercetin (Fig 7). SIRT1 promotes PGC-1α pathway activity [31–33], thus protein abundance of SIRT1 was measured and was 20% higher in dystrophic muscle regardless of treatment compared to healthy muscle. In contrast, SIRT1 activity was lower in dystrophic muscle as histone 3 lysine 9 acetylation (H3K9ac) was 80% higher in dystrophic muscle compared to control. Due to the deacetylase activity of active SIRT1 increased H3K9ac is indicative of decreased SIRT1 activity. Downstream pathway components and end products of the PGC-1α pathway were similar between all groups. Due to the potential function of quercetin as an AMPK activator [32, 34, 35], pAMPK T172 was measured to assess AMPK activity. We found that AMPK activity was lower by more than 40% in dystrophic muscle compared to control, and independent of feeding treatment.

## Discussion

Duchenne muscular dystrophy is a muscle wasting disease that leads to progressive deterioration of muscle function and muscle health. Muscle atrophy and increased muscle injury negatively impact motor function and eventually lead to death from cardiac or respiratory failure. Protection of muscle from secondary effects of dystrophin deficiency such as inflammation, metabolic dysfunction and free radical injury while simultaneously driving utrophin expression and mitochondrial biogenesis may slow disease progression and prolong muscle function. Up regulation of PGC-1α in dystrophic muscle is well recognized to maintain muscle function, decrease histological damage and increase utrophin protein abundance and localization [14–18]. Currently needed is a pragmatic strategy to translate this mechanistic understanding into clinical practice. As such, quercetin is a promising therapeutic supplement that exhibits both antioxidant and anti-inflammatory properties and also drives mitochondrial biogenesis and utrophin upregulation through PGC-1α activation. Supporting a rationale for clinical applicability, quercetin is commonly sold as an over-the-counter supplement and is readily available to the DMD community. Experimental evidence to support this postulate is derived from an initial investigation where we found that a 6 month dietary intervention with quercetin decreased histological injury in the dystrophic diaphragm [23] and heart [24]. In a subsequent 12-month study the beneficial effects following six months of treatment were recapitulated [25]. These effects appeared to be transient, however, in that control diaphragms from dystrophic mice were functionally, histologically, and biochemically indistinguishable from quercetin-treated diaphragms by the end of the study period. We hypothesized that advanced disease progression in the diaphragm may limit the therapeutic potential of quercetin and that the milder phenotype found in dystrophic limb muscles would be a more amenable intracellular environment to support the therapeutic effects of quercetin.

Similar to our previous findings in diaphragms [25] from the same animals used herein, most outcomes examined at the end of the treatment period were not responsive to the quercetin intervention. Of note, dietary quercetin consumption was associated with prevention of approximately 50% of the disease-related losses in specific tension and fatigue resistance in the soleus. Improved specific tension in the soleus is surprising considering histological parameters were not improved by dietary quercetin enrichment. Hence, muscle function was improved in quercetin-treated mdx mice compared to control mice despite similar degrees of muscle damage/unit cross sectional area. We encourage future studies intended to improve clarity regarding the broad therapeutic effects of this strategy.

While these functional benefits are cause for limited enthusiasm our biochemical and histological findings require caution. On the whole, transcript expression was similar between treated and untreated mdx mice and cellular functions probed in this investigation were largely suppressed compared to C57. Published studies indicate that quercetin drives the PGC-1α pathway through the deacetylase activity of SIRT1 [32, 36, 37] such that SIRT1 deacetylates PGC-1α and ultimately leads to mitochondrial biogenesis, increased utrophin abundance, a shift toward more oxidative fiber types, and decreased disease severity [14–18]. We previously noted quercetin insensitivity in diaphragm tissues marked by increased SIRT1 protein abundance coupled with impaired SIRT1 function [25]. In this investigation a similar mechanism is apparent despite continued quercetin supplementation. We proposed previously that decreased ATP content found in dystrophic muscle may be part of that mechanism by limiting the potential of quercetin to increase SIRT1 activity via a blunted ATP/cAMP/pSIRT1 pathway. Alternatively, we speculate that the lower abundance of NAD+ in dystrophic muscle [38], a cofactor of SIRT1 activation [39], may also limit the capacity of quercetin to drive SIRT1 activity. Intriguingly, in a short-term investigation, supplementation with nicotinamide riboside to augment the muscle NAD+ pool was sufficient to increase regeneration following cardiotoxin injection in dystrophic muscle [40]. Hence, we propose that the combination of quercetin, to increase SIRT1 activity, and nicotinamide riboside, to increase muscle NAD+ content in order to sustain increased SIRT1 activity, may provide long term therapeutic relief and greater impact than either approach applied independently and may overcome limitations in SIRT1 activation caused by age and/or disease severity.

Given the largely unremarkable histological and biochemical findings in this investigation, one of the most notable findings in the current study was the quercetin-mediated elevations in spontaneous activity in caged mice. It is unclear if quercetin directly impacts behavior such that it serves to stimulate activity via mechanisms beyond the scope of this investigation [41] or below our detection threshold, or if quercetin supports improved function such that it permits increased spontaneous activity. Nevertheless, that there was increased spontaneous physical activity provides an alternative interpretation of data contained herein. Increased physical activity may hasten the decline of dystrophic muscle [42–44]; hence, improved specific tension in the face of increased activity supports the potential therapeutic role of quercetin. This effect seems particularly profound in the diaphragm as increased spontaneous physical activity led to significantly impaired diaphragmatic function in animals of similar age [11]. Hence, that respiratory and diaphragmatic function and histopathology were not further impaired compared to untreated mdx mice in our previous investigation [25] may also be suggestive of a therapeutic success. These histopathological results were largely recapitulated in limb muscle in this investigation, aside from the potential of increased fibrosis detected via trichrome staining but not fibronectin immunohistochemistry. Given this integrated understanding from an established model of DMD, the current findings may be all the more important in the eventual translation of findings to clinical populations.

Comprehensive reports comparing dystrophic and healthy muscle from old animals, as performed currently, are rarely reported in the existing literature [45]. Absolute and relative muscle mass was increased in dystrophic muscle compared to age-matched C57 mice suggestive of pseudohypertrophy. Further, tetanic force was similar between groups but specific tension was greatly impaired in both the soleus and EDL at the end of the treatment periods indicating a compromised muscle quality in mdx mice. Consistent with previous reports, in dystrophic mice the soleus had compromised resistance to fatigue [43] and the EDL was more susceptible to contraction-induced injury compared to control [2]. Histologically, the dystrophic soleus had damage consistent with dystrophinopathy including increased centralized nuclei, necrotic area, immune cell infiltration, and fibrosis [46, 47]. Fiber area distribution was also consistent with dystrophin deficiency with a large proportion of small diameter fibers and much higher variability in fiber size resulting in similar mean fiber area compared to control [48]. Our biochemical evaluation found decreased transcript expression related to mitochondrial biogenesis, mitochondrial content, resistance to oxidative stress, and cellular stability.

In total, the results of this investigation are in agreement with our previous report of age-dependent quercetin insensitivity leading to underwhelming therapeutic effects by the conclusion of the study period. Specifically, quercetin insensitivity is made clear by a failure to increase SIRT1 activity and may be due to decreased ATP and/or NAD+ content in dystrophic muscle. Future investigations should combine quercetin with an agent to increase the NAD+ pool in order to maximize therapeutic benefits. Furthermore, this study underscores the importance of long-term investigations as therapeutics applied to the DMD community would be expected to be employed for years. Only after 8 months of treatment was the transient therapeutic nature of quercetin apparent in respiratory function [25]. Given the histological and biochemical data from the soleus improved function will likely be lost with advancing age. Data interpretation is complicated by our finding of increased spontaneous activity in quercetin-treated mice without widespread activity-induced tissue damage in the limb muscles. This latter finding highlights a novel experimental facet of this investigation and may hold implications for clinical translation.

## Supporting Information

S1 Table Complete Data Set. Individual mouse data for all measures including muscle function, histopathology and biochemistry.
(XLSX)
